# Elevated telomere dysfunction in cells containing the African-centric Pro47Ser cancer-risk variant of TP53

**DOI:** 10.18632/oncotarget.26980

**Published:** 2019-06-04

**Authors:** Stephen Tutton, Zhong Deng, Nitish Gulve, Olga Vladimirova, Kate Beishline, Andreas Wiedmer, Maureen Murphy, Paul M. Lieberman

**Affiliations:** ^1^ The Wistar Institute, Philadelphia, PA, USA; ^2^ Childrens Hospital of Philadelphia, Philadelphia, PA, USA; ^3^ Bloomsburg University of Pennsylvania, Bloomsburg, PA, USA

**Keywords:** TP53, telomere, TERRA, DNA damage, polymorphism

## Abstract

Subtelomeric transcription and chromatin can have a significant impact on telomere repeat maintenance and chromosome stability. We have previously found that tumor suppressor protein p53 (TP53) can bind to retrotransposon-like elements in a majority of human subtelomeres to regulate TERRA transcription and telomeric histone acetylation in response to DNA damage. TP53 also prevents the accumulation of γH2AX DNA-damage signaling at telomeres. We now show that the inherited TP53 polymorphism Pro47Ser (hereafter S47), which is enriched in populations of African descent, is associated with elevated marks of telomere dysfunction. We found that human and mouse cells carrying the S47 variant show increased γH2AX DNA-damage signals at telomeres, as well as reduced TERRA transcription and subtelomeric histone acetylation in response to DNA damage stress. Cell-lines containing inducible genes for P47 or S47 versions of p53, as well mouse embryo fibroblasts (MEFs) reconstituted with human p53, showed elevated telomere-induced DNA damage foci and metaphase telomere signal loss in cells with S47. Human lymphoblastoid cell lines (LCLs) derived from individuals homozygous for S47, show increased accumulation of subtelomeric γH2AX and unstable telomere repeats in response to DNA damage relative to age matched LCLs homozygous for P47. Furthermore, LCLs with S47 had reduced replicative lifespan. These studies indicate that the naturally occurring S47 variant of p53 can affect telomeric chromatin, telomere repeat stability, and replicative capacity. We discuss the potential evolutionary significance of the S47 variant to African populations with respect to telomere regulation and the implications for inherited health disparities.

## INTRODUCTION

The tumor suppressor p53 (TP53) is among the most frequently mutated genes in human cancers [1]. In addition to somatic mutations, p53 is also subject to naturally occurring variants due to single nucleotide polymorphisms (SNPs) that can alter p53 function and potential risk for cancer and related disease [2]. The Pro47Ser polymorphism (rs1800371) (referred to as S47) is present 1–2% of African Americans and 6-8% in certain African populations, but is largely undetected in Caucasian American populations [3]. The S47 variant has been shown to have functional consequences by attenuating p53 transcriptional activation and pro-apoptotic functions [4]. The S47 variant has also been shown to be resistant to cisplatin-induced cell death [5] and to fail to induce mitochondrial initiated cell death and ferroptosis [6]. More recently, tumor cells containing the S47 variant have been found to exhibit decreased mitochondrial function and increased Warburg metabolism [7].

A principal function of p53 is to protect the genome from excessive mutagenesis, degradation, and instability [8]. We have previously shown that p53 has a direct role in regulating the stability of human telomeres [9]. Telomeres are repetitive DNA elements that protect linear chromosomes from mistaken double strand break repair and chromosome instability [10, 11]. Telomere repeat stability is controlled, in part, by telomere repeat binding factors and telomeric chromatin [12, 13]. The chromosome regions adjacent to the telomere repeats are referred to as subtlomeres and can also contribute to telomeric chromatin and telomere repeat stability [14]. Subtelomeres can bind to sequence-specific factors and at least one third of human subtelomeres contain high-affinity, sequence-specific p53 binding sites within 10 kB of the telomere repeat track [9, 15]. Subtelomeric binding of p53 stimulates transcription of telomere repeat-encoded RNA (TERRA) and also induces histone acetylation throughout the subtelomeric and telomere repeat region in response to DNA damage signaling [9]. CRISPR/Cas9 deletion of a single p53 binding site in chromosome 18p resulted in the aberrant regulation of TERRA and subtelomeric transcription of PARD6G, as well as the aberrant accumulation of γH2AX DNA damage signaling at the same telomere in response to DNA damage stress [9]. Here, we investigate the impact of the S47 variant of p53 in telomere maintenance.

## RESULTS

### The S47 variant of p53 is compromised for telomeric transcription activation

**Figure 1:**
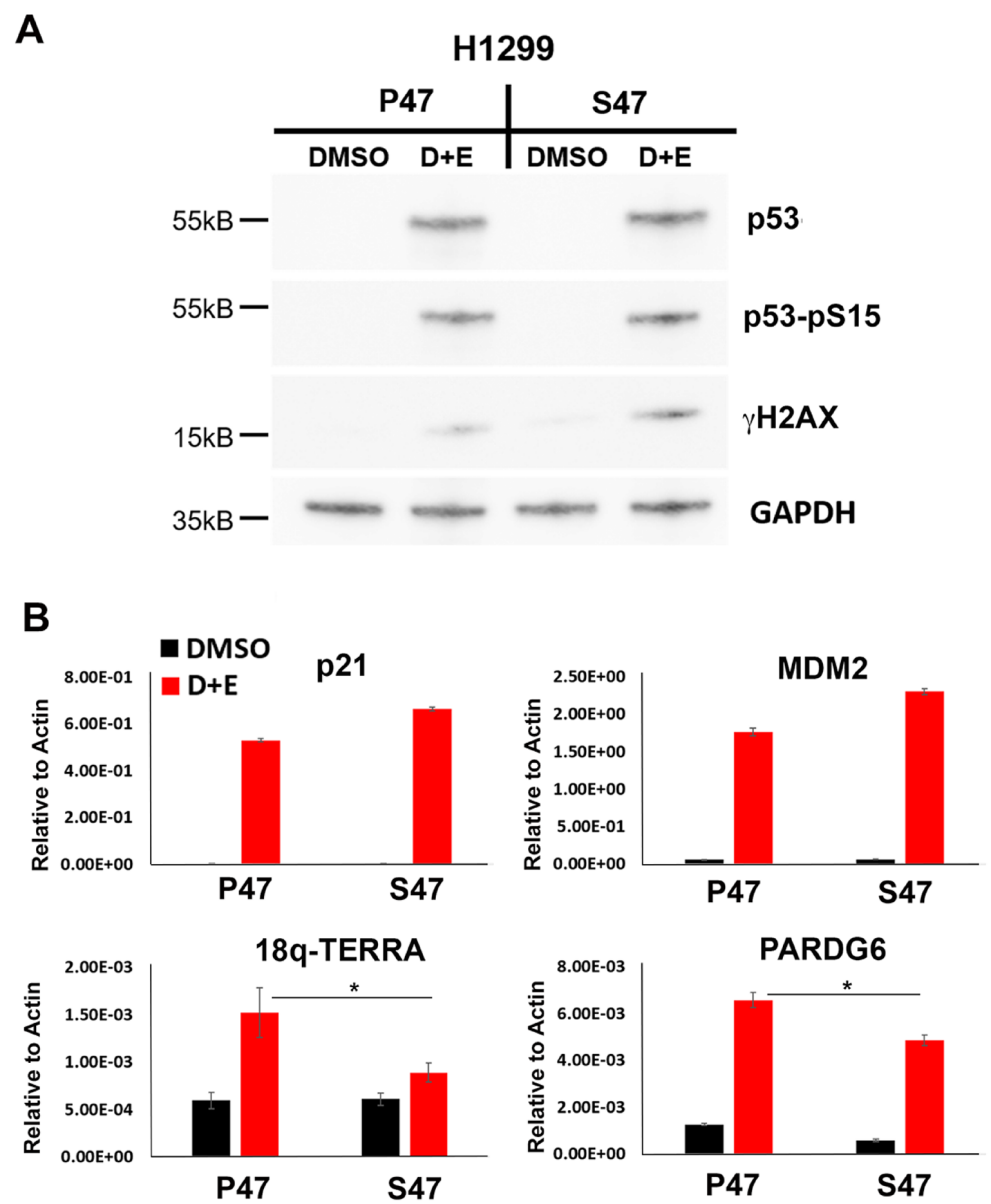
Human p53 S47 is deficient for DNA damage-induced activation of TERRA transcription. H1299 cells reconstituted with either P47 or S47 variant of p53 were treated with either DMSO or with doxycycline and etoposide (D+E) were assayed by (**A**) Western blot for expression of p53, p53-pS15, γH2AX or GAPDH, or (**B**) RT-qPCR for p21, MDM2, 18q-TERRA, or PARD6G relative to Actin control. Error bars represent SD. ^*^p < .05. *t*-test.

To investigate the function of S47 in human cells, we utilized p53 null H1299 cancer cell lines that were reconstituted with doxycycline-inducible p53 genes carrying either WT p53 (P47) or the S47 variant. P53 was induced and activated by addition of both doxycycline and 1 μM etoposide for 24 hrs to induce DNA damage signaling (Figure 1). After 24 hrs of p53 induction cells were assayed by Western blot for total p53 and Ser15 phosphorylated p53 (p53-pS15) (Figure 1A). We found that nearly identical levels of p53 and p53-pS15 were induced for P47 and S47, along with a modest increase in total levels of γH2AX in the S47 cells relative to P47 (Figure 1A). Using the same cells and conditions, we next analyzed p53-dependent transcription by RT-qPCR (Figure 1B). We found that the well-characterized p53 response genes p21 and MDM were both activated to similar or higher levels in cells expressing S47 relative to P47. In contrast, induction of the 18q TERRA transcript was markedly reduced (~70%) and the 18q subtelomeric gene PARD6G was partially reduced in cells expressing S47 relative to P47 (Figure 1B). This suggests that the telomeric transcription activation function of p53 is selectively compromised in the S47 variant.

### Elevated telomeric γH2AX in S47 expressing H1299 cells

**Figure 2:**
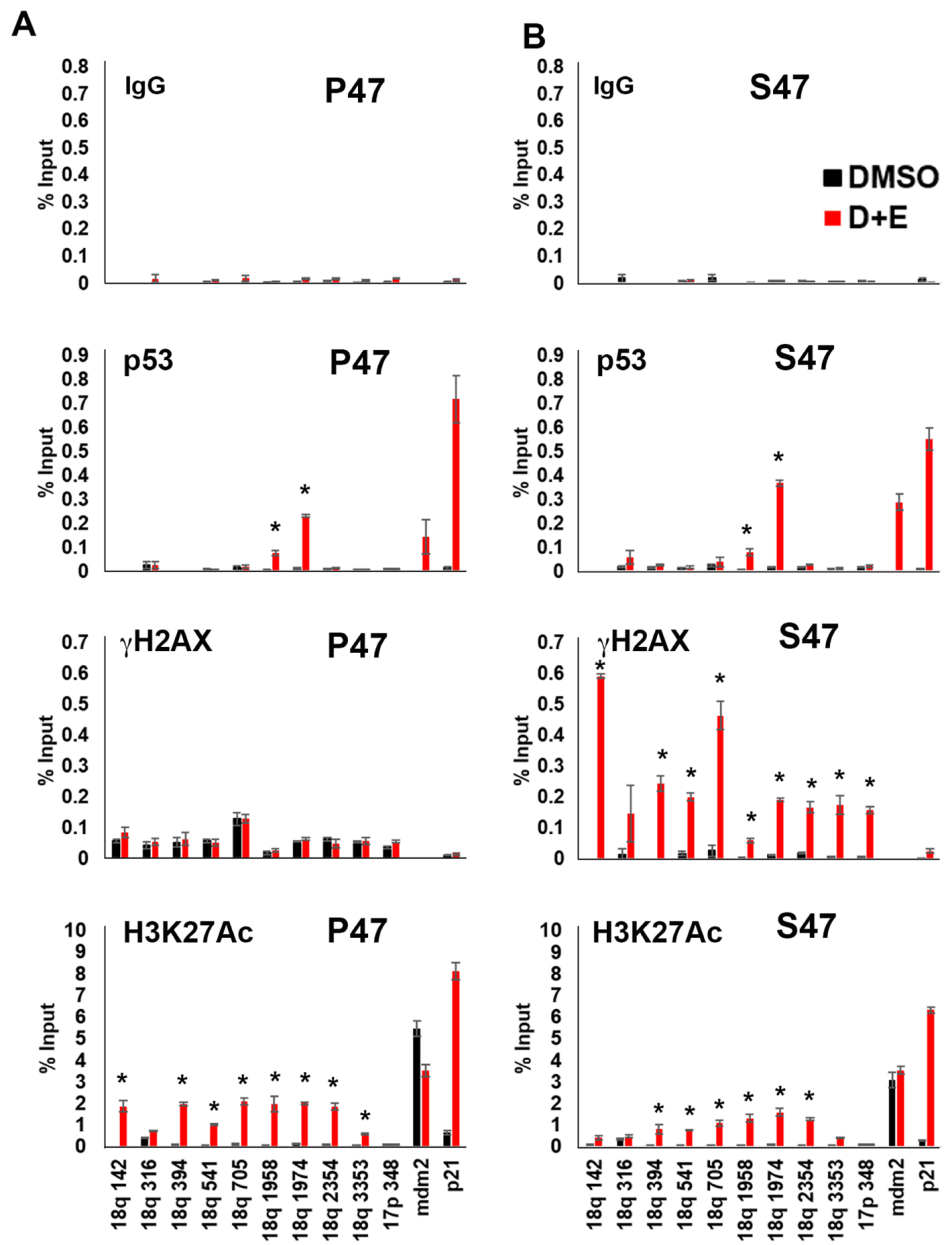
Human p53 S47 is deficient for DNA damage-induced telomere histone acetylation and suppression of γH2AX accumulation. H1299 cells reconstituted with either P47 (panel **A**) or S47 (panel **B**) variant of p53 were treated with either DMSO or with doxycycline and etoposide (D+E) were assayed by ChIP for IgG, p53, γH2AX, H3K27Ac at various positions across the 18q subtelomere (numbers are relative bp from the telomere repeats), or the p53 binding sites in the mdm2 or p21 promoter. Error bars represent SD. ^*^p < .05 *t*-test.

H1299 cells treated as described above (Figure 1) were analyzed by Chromatin Immunoprecipitation (ChIP) assay for binding of p53, γH2AX and H3K27Ac at various positions along the 18q subtelomere and at the p53-binding sites associated with the mdm2 and p21 genes (Figure 2). ChIP with p53 antibody revealed a strong p53 binding induced by doxycycline and etoposide at the p21 and mdm2 sites. Similar p53 binding was observed at the 18q 1974 primer position (located 1974 bp from the telomere repeat junction). The S47 variant of p53 bound as well or better than P47 to the subtelomeric DNA sites. γH2AX ChIP revealed that cells expressing S47 showed consistently increased levels of γH2AX across the entire 18q subtelomere, but no detectable increase was observed in P47 expressing H1299 cells. In contrast, H3K27Ac was induced to higher levels in subtelomere regions adjacent to terminal repeats (18q142) in P47 relative to S47 (Figure 2). These findings indicate that the S47 variant of p53 binds adequately to the sub-telomere but fails to induce high levels of H3K27Ac and prevent the enrichment of γH2AX at a p53-responsive subtelomere.

### Reduced telomeric DNA synthesis and G0 cell cycle arrest in response to DNA damage stress in S47 expressing H1299 cells

**Figure 3:**
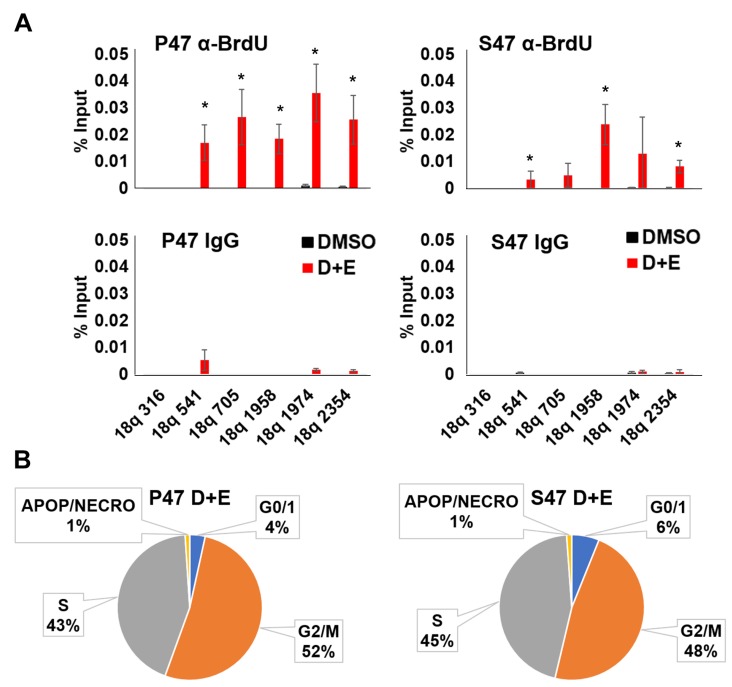
Human p53 S47 is deficient for telomeric DNA repair synthesis. H1299 cells reconstituted with Doxycycline-inducible P47 or S47 variant of p53 were treated with either DMSO or with doxycycline and etoposide (D+E) and then assayed by (**A**) BrdU incorporation followed by DNA-IP with anti-BrdU (top) or IgG (lower) and assayed by qPCR at various positions across the 18q subtelomere. ^*^p < .05 *t*-test. (**B**) FACS cell cycle analysis of H1299 cells treated as indicated in panel A above.

To determine if DNA replication and/or DNA repair are impaired in S47 expressing H1299 cells, we assayed BrdU incorporation by DNA-IP (DIP) assay. Cells were treated with doxycycline and etoposide as above to induce p53 and DNA damage, followed by addition of BrdU and 24 hr recovery time. The BrdU-DIP assay was used to measure the rate of DNA synthesis at the 18q subtelomere. We focused on the 18q subtelomere since it was found to selectively accumulate γH2AX in S47, but not P47 expressing cells (Figure 2). The BrdU-DIP assay revealed that replication and/or repair was elevated in P47 cells, relative to S47 (Figure 3A). Cell cycle analysis indicated that S47 cells had a greater population in G0, suggesting that fewer cells re-entered the cell cycle after p53 and DNA damage induction (Figure 3B). Taken together, these findings suggest that S47 expressing cells are less efficient at limiting and/or repairing DNA damage at p53-responsive subtelomeres.

### MEFs with human p53 S47 are impaired for DNA damage-induced TERRA expression.

**Figure 4:**
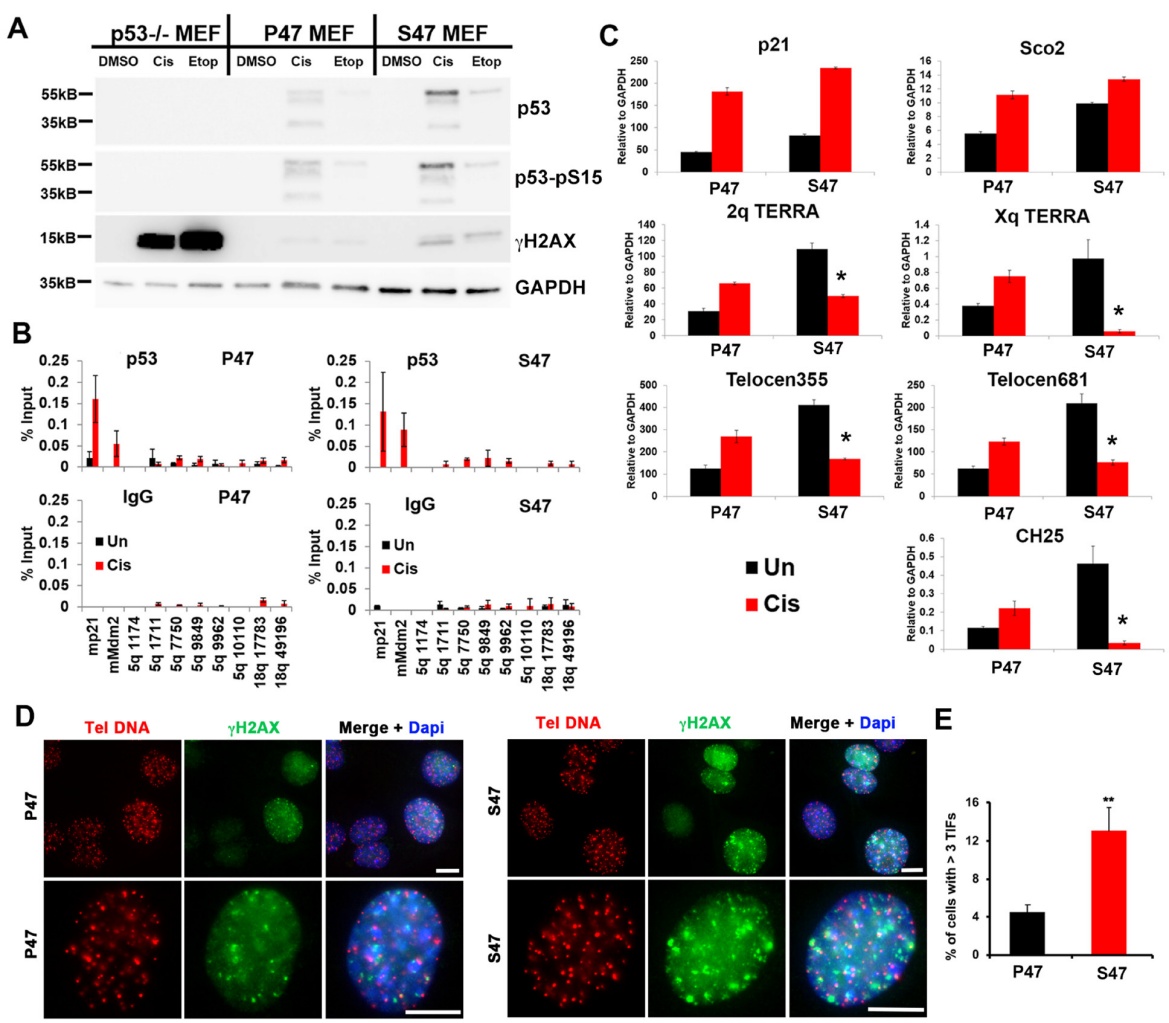
MEFs reconstituted with human p53 S47 fail to induce mouse TERRA in response to DNA damage stress. (**A**) p53–/– MEFs reconstituted with human p53 P47 or S47 were treated with either DMSO, cisplatin (Cis), or etoposide (Etop) and assayed by Western blot for p53, p53-pS15, γH2AX, or GAPDH, (**B**) p53–/– MEFs described in panel A treated with either DMSO or cisplatin (Cis) were assayed by ChIP for p53 or IgG at mouse p53 binding sites in mp21, mMdm2, or various positions across the 5q subtelomere. (**C**) p53–/– MEFs described in panel A treated with either DMSO or cisplatin (Cis) were assayed by RT-qPCR for p21, Sco2, and various subtelomeric positions for chromosome specific TERRA RNA. (**D**) TIF assay for p47 (left) or S47 (right) MEFs using Tel C PNA FISH probe (red) or γH2AX antibody for IF (green) and Dapi (blue). Upper panels at 20x and lower panels at 60x magnification. Scale bar=5 μM. (**E**) Quantification of TIFs shown in panel D. ^**^p < .01.

To corroborate the results above, we took advantage of mouse embryo fibroblasts (MEFs) isolated from P47 and S47 mice with knock-in humanized p53 [6]. We used this system to assay the effect of p53 on the cellular and telomeric response to DNA damaging agents cisplatin (Cis) or etoposide (Etop). We found that cisplatin or etoposide treatment led to a robust activation of γH2AX in p53–/– cells, while this effect was strongly diminished by P47, and to a lesser extent by S47 expressing MEFs (Figure 4A). ChIP-qPCR revealed that p53 binding sites at the murine CDKN1A (p21) and Mdm2 genes bound to P47 and S47 p53 with similar enrichments (Figure 4B). We next analyzed p53 binding sites at sub-telomeres, which were identified based on previous ChIP-Seq data analyses [9]. We observed weak binding of p53 at the subtelomeres, with higher signals emerging for S47 (Figure 4B). RNA transcripts measured by RT-PCR revealed that cisplatin induced high level transcription activation for p21 in both P47 and S47. The relative induction for Sco2, a cytochrome C oxidase assembly factor previously found to be differentially responsive to S47, was reduced for S47, as expected [5, 6]. We found that cisplatin-induced TERRA transcription from mouse subtelomeres at 2q, Xq, Telocen355, Telocen681, and CH25 were severely compromised in S47 MEFs relative to P47 expressing MEFs (Figure 4C). We next analyzed these MEFs for telomere-induced DNA-damage foci (TIFs) by immunofluorescence assay (IF) colocalization of γH2AX with telomere DNA FISH signal (Figure 4D and 4E). We found that S47 MEFs have ~3 fold increase in cells with 3 or more TIFs relative to P47 (Figure 4E). These findings indicate that the S47 variant is defective at inducing TERRA expression and reducing γH2AX and telomere-associated DNA damage in mouse MEFs, similar to the case in human tetracycline-inducible cells.

### Human lymphoblastoid cell lines (LCLs) with S47 show increased telomere DNA damage and reduced TERRA expression.

**Figure 5:**
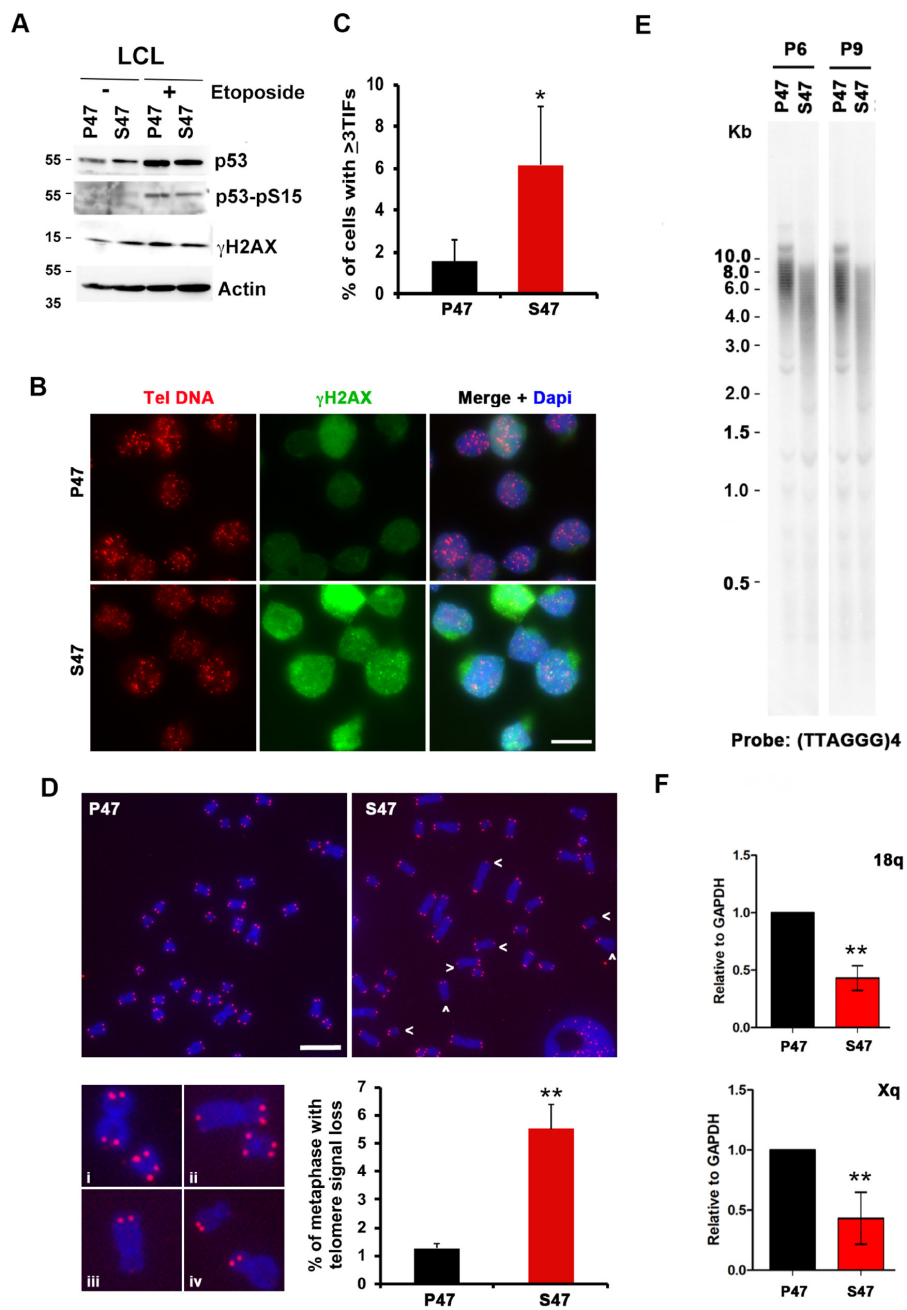
Telomere DNA damage foci and decreased TERRA in human LCLs carrying homozygous p53 S47. Human LCL carrying with WT or S47 homozygous p53 were assayed by (**A**) Western blot for p53, p53-pS15, γH2AX, and Actin, (**B**–**C**) IF for telomere DNA (red), γH2AX (green) and Dapi (blue), and quantified for percentage of TIFs per cell, (**D**) metaphase chromosome telomere FISH with TelC PNA probe (red) and DAPI (blue), and (**E**) telomere length assay for passages P6 and P9, and (**F**) RT-qPCR for TERRA transcripts at 18q or Xq. Error bar represents SD and *p*-values determined by chi-square (TIF assay). ^**^p <. 01.

EBV immortalized lymphoblastoid cell lines (LCLs) containing either homozygous P47 or S47, obtained from Coriell Institute, were described previously [6]. Western blot analysis revealed similar levels of p53, phosphorylated p53 (p53-pS15), and γH2AX in untreated and etoposide treated cells (Figure 5A). Immunofluorescence analysis revealed the accumulation of γH2AX foci with a ~3 fold higher rate of colocalization at telomere DNA in S47 cells compared to P47 cells (Figure 5B and 5C). Telomere integrity was further examined by metaphase FISH (Figure 5D). We found that telomere signal loss was elevated ~5 fold in S47 relative to P47 LCLs (Figure 5D). We next assayed average telomere length by Southern blot analysis (Figure 5E). We observed a significant reduction in average telomere length in S47 relative to P47 at two different passages P6 and P9 (Figure 5E). We next assayed the expression of TERRA at the 18q and Xq subtelomeres and found a reduction in TERRA transcription in S47 compared to P47 LCLs (Figure 5F). These findings indicate that human LCLs carrying the S47 variant show increased γH2AX-positive TIFs, loss of metaphase telomere signal, decreased telomere length, and decreased expression of TERRA expression relative to the more common variant P47.

## DISCUSSION

p53 is known to play a critical role in telomere regulation through a telomere-shelterin-ATM/ATR-p53 axis [12, 16]. Loss of shelterin binding to telomeres exposes telomere DNA to double strand break recognition sensing and DNA damage signaling pathways. Loss of function mutations in p53 permit the accumulation of critically short and uncapped telomeres and consequent chromosome instability observed in many p53-mutated cancers. In addition to this canonical pathway, we describe a reciprocal p53-telomere regulation axis mediated through the direct binding of p53 to sequence-specific sites in human and mouse subtelomeres [9, 15].

Direct binding of p53 to subtelomeres can alter telomere function through several mechanisms. p53 can induce TERRA transcription [9, 17]. Direct binding of p53 can induce telomeric histone acetylation [9]. p53 binding, TERRA transcription, and histone acetylation correlate with telomere end-protection as measured by the reduction in γH2AX accumulation and increased DNA damage repair synthesis at telomere repeats [9]. A CRISPR/Cas9 engineered disruption of the 18q TERRA promoter demonstrated that TERRA transcription is important for telomere DNA synthesis and repair, especially under conditions of replication stress [18]. p53 binding may also contribute to the expression of telomere DNA damage response RNAs (tDDRNAs) that contribute to telomere end-protection and genome stability [19]. Thus, p53 can provide a direct and local protective response to DNA damage stress through sequence-specific p53 bound sites and the regulation of proximal transcription and histone modifications.

p53 mutations and naturally occurring variants may alter site-specific functions of p53, including those at telomeres. Loss of function mutations found in cancers likely provide a permissive environment for telomere shortening and dysfunction in proliferating cells, a condition that facilitates genomic instability. However, it is not known how gain-of-function mutations and natural variants of p53 may alter telomere length and regulation. Here, we have found that cells harboring the S47 variant of p53 have dysfunctional telomere regulation in human and mouse cell culture models. In each model, we found that S47 impaired telomere transcription and DNA integrity. In human cells, we found that S47 increased the formation of telomeric histone H3K27Ac and decreased accumulation of γH2AX. Increased histone acetylation is likely due to the direct recruitment of histone acetylases by p53 at sequence-specific subtelomeric DNA binding sites. The accumulation of γH2AX is likely due to the reduced efficiency of S47 in DNA damage repair at telomeres. This effect on telomere DNA damage repair is likely due to the direct effect of p53 on TERRA transcription and histone acetylation. However, it is also possible that indirect, non-telomeric functions of p53 also contribute to telomere dysregulation observed with S47 variant.

Other mutations in p53 have been shown to affect telomere structure and regulation. A dominant negative mutation of p53 (N340Q/L344R) was found to reduce TERRA expression and enhance hepatocellular carcinoma [20]. p53 mutation and disruption correlated with shorter mean telomere lengths in chronic lymphocytic leukemias [21, 22]. Mutations that lead to constitutive activation of p53 resulted in telomere shortening and telomere dysfunction, in part due a p53-mediated repression of shelterin proteins [23, 24]. Thus, p53 mutations may have complex and indirect effects on telomere regulation, in addition to its direct binding at subtelomeres.

Natural genetic polymorphisms may contribute to population health disparities. The S47 variant of p53 has been shown to occur predominantly in individuals of African descent [5, 6, 25]. Although the S47 allele is relatively rare and epidemiological studies are limited, this variant has been shown to correlate with increased pre-menopausal breast cancer risk in African American women [25]. While S47 has several phenotypes, including altered mitochondrial function [7], it is possible that its cancer risk is linked to its effects on telomere regulation. LCLs with homozygous S47 showed accumulation of telomere DNA damage, telomere length shortening, and reduced replicative life span. Telomere shortening and DNA damage accumulation are important drivers of replicative senescence [26, 27]. Inherited telomere length variations have potential impact on health and life-span disparity [28]. An inverse correlation between age and telomere length has been well-documented [29]. Telomere length has also been shown to vary based on geographical origin [30]. Average telomere lengths have been found to be longer among African populations relative to European [30]. One speculation is that longer telomere lengths provides some immunological advantage, potentially by enhancing replicative capacity for rapidly expanding B and T-cells in response to chronic infection [31]. This immunological advantage may be at the expense of increased cancer risk through a reduced tumor suppressor function and increased replicative life span for somatic cells. To date, no single gene variant has been strongly associated with telomere-length differences [32]. Our studies in LCLs suggests that the S47 variant results in shorter, unstable telomeres with increased sensitivity to DNA damage and reduced replicative senescence. This is in contrast to what might be expected for African populations with longer telomeres. However, the African-centric S47 allele is relatively rare, found only in 10% of African populations and 1.2% of African Americans. Therefore, it is possible that S47 compensates for inherited longer telomere length, with a cost of increased telomere instability and decreased replicative capacity.

## MATERIALS AND METHODS

### Cells and plasmid DNA

HCT116 +/+ and −/− cells were generously contributed by Bert Vogelstein’s laboratory (Johns Hopkins School of Medicine). HCT116 cells were cultured in Dulbecco’s Modification of Eagle’s Medium (DMEM, Cellgro, 10-027) with 10% fetal bovine serum (FBS), 1% Pen/Strep and 4.5 g/L glucose and L-glutamine. H1299 tet-inducible p53 cell lines were cultured in DMEM, 1% Pen/Strep and with Tet-system approved 10% FBS (Clontech, 631106). A concentration of 0.75 ug/ml doxycycline was used to induce p53. Etoposide (Sigma) 50 mM in DMSO was added to cells, at 1, 2, or 50 μM final concentration for 24 hr or times indicated. MEFs was P47 and S47 mice, as well as LCLs from individuals homozygous for P47 and S47, were previously described [6].

### ChIP-qPCR and BrdU-IP

ChIP-qPCR was performed essentially as described previously [33]. All qPCR primers have been described previously [9]. BrdU-IP was described previously [18].

### Antibodies

The following antibodies were used: mouse Anti-p53 (Ab6) (Calbiochem OP43); rabbit anti-phospho-p53 (Ser15) (Cell Signaling 9284); mouse anti-phospho-Histone H2A.X (Ser139) (Millipore 05-636); rabbit anti-Histone H2A.X (Millipore 07-627); rabbit anti-acetyl-Histone H3 (Lys9) (Millipore 07-352); rabbit anti-Histone H3 (K4me1) (abcam ab8895); rabbit anti-Histone H3 (K27Ac) (abcam ab4729); rabbit anti-Histone H3, CT, pan (Millipore 07-690); rabbit anti-GAPDH (14C10) (Cell Signaling 2118).

### RT-qPCR

RNA was isolated from cells by suspending well in 1 ml Trizol (Life Technnologies) at RT. 200 μL chloroform was added, and the sample shaken by hand for 15 sec. After 2–3 min at RT, samples were centrifuged at 12,000 × G for 15min at 4° C. 550 μL of the upper aqueous phase was transferred to a new eppendorf, mixed with 500 μL isopropanol, and incubated at RT for 10 min. RNA is precipitated at 12,000 × g for 10 min at 4° C, washed in 75% ethanol, dried briefly and dissolved in DNAse I reaction mix prepared in DEPC-treated water. DNAse I treatment proceeds at 37° C for 45 min, then inactivated at 65° C for 5 min. RNA was reverse transcribed with random primers using iScript reverse transcription supermix (Biorad) or for TERRA transcript detection a gene specific primer (GSP) designed to complement telomere repeats (ccctaaccctaaccctaaccctaaccctaa) and SuperScript III reverse transcriptase (Invitrogen). cDNA was quantified by qPCR using the ΔΔC_t_ method using Power SYBR Green (Applied Biosystems) and the primers have been described previously [9]. Primers for human and mouse telomeres 2q, Xq, Telocen355, Telocen681, and CH25 were described previously [9, 33, 34].

### Telomere length and microscopy analysis

Telomere length, TIF and metaphase telomere signal intensity assays were performed as described previously [35].

### Statistics

Error bars indicate standard deviation. *P*-values were calculated by two-tailed student *t*-test with at least three replicates.

## Abbreviations

p53: TP53
WT: wild-type
TERRA: Telomere Encoded Repeat RNA
S47: p53 Pro47Ser polymorphism
MEFs: mouse embryo fibroblasts
LCLs: lymphoblastoid cell lines
ChIP: chromatin immunoprecipitation
RT-qPCR: reverse transcription-quantitative polymerase chain reaction
TIF: telomere induced DNA damage foci
IF: immunofluorescence
FISH: fluorescence in-situ hybridization
Cis: cisplatin
Etop: etoposide
